# The role of surgical resection in patients with brain metastases

**DOI:** 10.3332/ecancer.2013.308

**Published:** 2013-04-18

**Authors:** Mustafa Aziz Hatiboglu, David M Wildrick, Raymond Sawaya

**Affiliations:** 1 Department of Neurosurgery, Kocaeli Derince Training and Research Hospital, Kocaeli, Turkey; 2 Department of Neurosurgery, The University of Texas MD Anderson Cancer Center, 1515 Holcombe Blvd., Houston, TX, USA

**Keywords:** brain and nervous system, complications, radiotherapy, surgery, control, survivorships and outcomes

## Abstract

Brain metastasis is a devastating complication of systemic malignancy that affects a considerable number of cancer patients. The appearance of brain metastases is often considered to be a sign of poor prognosis; in patients with brain metastases poor survival time has been reported in the literature. Therefore, treatment of these brain lesions in cancer patients is important for quality of life, providing local tumour control, preventing death from neurological causes, and improving survival, although potentially only in a minority of patients. Surgical resection of brain metastases has been the cornerstone treatment in select patients. Careful patient selection, the use of appropriate surgical techniques, and surgical adjuncts are the major determinants of favourable outcome in patients undergoing resection of brain metastases. In this review, we explain the role of surgical resection in the treatment of patients with brain metastases with consideration of patient selection, surgical techniques and the use of intraoperative adjuncts.

## Introduction

Approximately 1.4 million people are diagnosed with cancer, and over half a million of them develop brain metastases in the USA every year [1]. The occurrence of brain metastases is an indication of poor outcome and is often considered to be the terminal stage in a patient with a systemic malignancy [2–4]. Given this fact, considerable efforts are provided for such patients, mainly to improve the quality of life by enhancing neurological functions, to improve survival and to prevent brain lesion-related mortality.

Without any treatment, the expected survival time of brain metastasis patients is about one month [3–5]. In the mid-1950s, whole-brain radiation therapy (WBRT) was first reported for treatment of brain metastases, and it improved survival from one month to 3–6 months [3–6]. The use of WBRT became standard of care in the 1980s, and it has remained a fundamental treatment for patients with brain metastases for a long time. Management strategies for brain metastases have evolved significantly during the last two decades owing to the advancements in new techniques and technology in the neurosurgical field. Moreover, with new imaging modalities, including computed tomography, and more recently, magnetic resonance imaging (MRI), surgical resection of brain metastases has become more popular.

A number of retrospective studies in the 1980s revealed the survival benefit of surgical resection in patients, especially those with a single, accessible brain lesion, with good functional status and with absent or controlled systemic disease [7–10]. To validate the effectiveness of surgical resection, in the early 1990s, two randomised clinical trials were performed to compare WBRT alone , against the combination of WBRT plus surgical resection, in patients with a single and accessible brain metastasis [11, 12]. These studies included patients with a single metastasis and mainly those with controlled or absent systemic disease. The patients showed a survival benefit of surgical resection followed by WBRT over WBRT alone (Table 1). After this validation of a survival benefit from surgical resection of brain metastases, surgical resection has become the cornerstone treatment for patients with brain metastases.

Stereotactic radiosurgery (SRS), which delivers a high dose of radiation to a discrete lesion of 3–3.5 cm in maximum diameter, has been introduced for treatment of patients with brain metastases [13]. Primarily patients with surgically inaccessible brain lesions, with active systemic cancer or with surgical comorbidities are candidates for SRS. Metastases in deep locations such as the thalamus, basal ganglia, and brainstem can be treated by SRS with somewhat less risk than with surgery [14]. To demonstrate the effectiveness of SRS for brain metastases, a study by the Radiation Therapy Oncology Group (RTOG), RTOG 9508, has shown a survival benefit of SRS plus WBRT over WBRT alone (6.5 months versus 4.9 months, respectively; *P*=0.0393) in patients with single brain metastases. This finding has significantly contributed to an increase in the use of SRS for treatment of brain metastases during the last decade [15].

Advances in new radiation therapy modalities allow new treatment options for brain metastases. However, surgical resection still remains the primary treatment option for patients with brain metastases. The purpose of this review is to explain the role of surgical resection in the treatment of patients with brain metastases with respect to patient selection, surgical techniques, and the use of intraoperative adjuncts.

## Rationale of Surgery for Brain Metastases

Surgical resection is recommended mainly for patients with a single brain metastasis in an accessible location, especially when the tumour size is large and causing a considerable mass effect or obstructive hydrocephalus. Surgery is also favoured in patients with good performance status, who are functionally independent (for example, spending less than 50% of time in bed), and in whom systemic disease is limited or absent [16]. Conversely, there is not enough evidence to favour surgical resection in patients with multiple brain metastases (more than 4 lesions), poor prognostic factors, and active or uncontrolled systemic cancer [2, 16]. Nevertheless, controlled systemic disease is not a requirement for aggressive surgical resection. Surgery may be preferred in some circumstances: (1) patients with controllable extracranial disease at one site, for example, having only bone metastases from breast cancer or lung cancer; (2) patients with a radioresistant primary cancer (e.g., renal cancer or melanoma); (3) patients with a large brain lesion causing mass effect or peritumoural oedema, although if there are multiple brain metastases, surgical resection is recommended for the dominant lesion [17, 18]; and (4) biopsy or surgical resection may be required to differentiate tumour necrosis from tumour recurrence in patients previously treated with radiation therapy. In contrast, surgical resection is not generally recommended for relatively radiosensitive tumour types such as small cell lung cancer, germ cell tumours or leukaemia and lymphoma [16]. They are more likely to be treated with WBRT alone once the diagnosis is made.

Although the role of surgical resection of a single brain metastasis has been well defined with prospective and several retrospective studies in the literature, its role for multiple brain metastases remains controversial, and no prospective randomised clinical trial has been performed yet comparing resection of single versus multiple brain metastases [11, 12, 19, 20]. The prognosis of patients with multiple brain metastases has been considered to be unfavourable, and such patients were considered to be poor candidates for resection owing to their expected shorter survival time [17, 21]. However, advances in surgical techniques and intraoperative technologies resulting in more aggressive and safer surgical resections have facilitated surgery for multiple brain metastases.

A few retrospective studies have shown the efficacy of surgical resection for multiple brain metastases [17, 18, 22, 23]. Paek *et al *[17] suggested that surgical resection of the dominant lesion among two or three lesions along with WBRT provided the same survival rate as is seen with resection of a single lesion in patients with brain metastasis. In a retrospective study from the University of Texas, MD Anderson Cancer Center (MD Anderson), Bindal *et al *[22] evaluated 56 patients who underwent surgical resection for multiple brain metastases (the maximum was three lesions). The patients were separated into two groups. Not all lesions were resected in the first group, and all lesions were resected in the second group. These patients were compared with a third group of 26 matched patients who underwent surgical resection of a single metastasis. Their results showed that the median survival time was 14 months for patients in both the second and third groups and only six months for those in the first group. Moreover, the risk of morbidity and mortality did not differ among the groups.

Wronski *et al *[23] did not find a significant outcome difference in patients who underwent surgical resection for single or multiple brain metastases. Similarly, Iwadate and colleagues [24] reported that there was no statistically significant survival difference after surgical resection between patients with a single metastasis and those with multiple metastases (8.7 months versus 9.2 months, respectively). Also, surgical reduction of tumours larger than 2-cm in maximum diameter was found to improve the efficacy of adjuvant radiation and to contribute to survival. They concluded that surgical resection of multiple brain metastases can improve the survival and enhance the quality of life in select patients.

The role of surgical resection for recurrent brain metastases has been studied as well. Al-Zabin and his colleagues [25] analysed 25 patients with recurrent solitary brain metastasis from lung cancer (84% with non-small cell lung cancer and 14% with small cell lung cancer). They showed significant functional improvement after surgical resection of recurrent brain metastases, and surgery provided these patients with a better quality of life despite their expected short survival time. Also, the multivariate analysis showed that the interval from cancer diagnosis to first brain metastasis and the interval between first and recurrent metastases were both significantly associated with survival. Bindal *et al *[26] reported 48 patients who underwent surgical resection of recurrent brain metastases. The median time between first craniotomy and diagnosis of recurrence was 6.7 months. The median survival time was 11.5 months after reoperation. Multivariate analysis showed that the presence of active systemic disease, a Karnofsky Performance Scale (KPS) score [27] of 70 or less (Table 2), a time to recurrence of less than four months, age ≥40 years, and a primary tumour type of melanoma or breast cancer were associated with poor survival. They also concluded that the first reoperation, as well as the second reoperation, can increase the survival time and quality of life. Arbit *et al *[28] also reported 214 patients with brain metastases from non-small cell lung cancer who underwent surgical resection. They concluded that reoperation is effective in prolonging survival for patients with recurrent brain metastases from non-small cell lung cancer. Although there is no prospective trial assessing the role of reoperation for recurrent brain metastases, these studies suggested a benefit for surgical resection of recurrent brain metastases in select patients.

The clinician should carefully evaluate patients with brain metastases who undergo surgical resection. Some advantages of resection should be kept in mind: (1) provides histological diagnosis, which is crucial for planning further treatment; (2) avoids long-term steroid use; (3) results in immediate improvement of intracranial mass effect and recovery of neurologic deficit or seizure; and (4) provides tissue samples for scientific purposes. Also, the appearance of a brain metastasis is the first sign of a neoplasm in approximately 10% of cancer patients. Surgery is helpful in making a diagnosis if no primary tumour can be found. Moreover, histological analysis may demonstrate a primary brain tumour or an abscess after resection of a brain lesion in a patient with systemic cancer [11].

## SRS for Brain Metastases

SRS is a less invasive local treatment and has the ability to spare the healthy brain tissue while treating the tumour. SRS is recommended for multiple brain metastases, lesions in locations not amenable to surgical resection, lesions less than 3–3.5 cm in maximum diameter, and for patients with active systemic disease or with other comorbidities preventing a surgical intervention. Another advantage of SRS is that radioresistant tumours such as renal cell carcinoma and melanoma respond to SRS. SRS provides an effective treatment option for brain metastases with acceptable results in terms of survival time and local tumour control rate. Previous studies have reported median survival times of 7.5–12.5 months [19, 29–32], one year local tumour control rates of 64–97% [29, 32, 33] and crude local tumour control rates of 71–95% [14, 31, 34].

## Surgical resection or SRS

Although SRS has emerged as a less invasive, but effective treatment for patients with brain metastases and thus is thought be an alternative to surgical resection, a debate has started on which treatment, surgical resection or SRS, is better for this group of patients, especially with a single lesion of less than 3.5 cm in maximum diameter. Several researchers have tried to determine which treatment is superior, but no convincing results have been found and controversy still exists. One prospective study by Muacevic and his colleagues [32], which was closed prematurely due to insufficient patient accrual, has compared SRS with surgical resection followed by WBRT. Sixty-four (of the planned 242) adult patients with a KPS score ≥70 and single, operable brain metastases ≤3 cm in maximal diameter were included in this multicentre, randomised controlled trial. Thirty-one patients were in the SRS arm and 33 were in the surgery + WBRT arm. Overall survival time was not significantly different between two arms. The median survival time was 10.3 months in the SRS group and 9.5 months in the surgery + WBRT group. Duration of freedom from local recurrence did not differ significantly between two groups. The one-year local tumour control rate was 96.8% in the SRS group and 82% in the surgery + WBRT group. However, the rate of recurrence at distant brain sites was found to be higher in the SRS group than in the surgery + WBRT group (26% and 3%, respectively). There was no difference between the two groups in terms of deaths from neurological causes. Similarly, two retrospective studies comparing SRS with surgical resection + WBRT have demonstrated that there was no survival difference between the two treatment groups [29, 33]. Also, there have been retrospective studies comparing SRS with surgical resection, revealing no statistical difference between the two groups [30, 31, 34]. Conversely, in a retrospective study by Bindal *et al *[19], including 62 patients in the surgical resection + WBRT group and 31 in the SRS + WBRT group, the median survival time in the surgery group was significantly longer than in the SRS group (16.4 months versus 7.5 months). Also, they showed a higher incidence of death from neurological causes in the SRS group than in the surgery group (50% and 19%, respectively). Although there is no prospective randomised clinical trial comparing surgical resection with SRS for brain metastasis treatment, and conflicting data do exist. Class II evidence suggests that better outcomes may be obtained with surgical resection for lesions larger than 3-cm in maximum diameter or those causing mass effect/midline shift, whereas SRS is recommended for surgically inaccessible, single lesions smaller than 3-cm in maximum diameter [16].

## Whole-brain radiation therapy after surgical resection

Surgery plus WBRT has been shown to be superior to WBRT alone in terms of survival, and it became a standard treatment for a single brain metastasis. Nonetheless, the question remains as to whether WBRT is necessary after surgical resection. To address this issue, Patchell *et al *[35] performed a prospective randomised clinical trial to compare surgery alone with surgery + postoperative WBRT as the initial treatment in patients with single brain metastases. This study included adult patients with a KPS score ≥70, who underwent complete resection of the single brain metastasis. Ninety-five patients were randomised into two groups after surgical resection with 49 patients assigned to postoperative WBRT and 46 patients to observation alone. The primary outcome was tumour recurrence in the brain. The recurrence rate in the surgery alone group was higher than in the surgery + WBRT group at the original tumour site (46% and 10%, respectively; *P*<0.001) and at distant sites (37% and 14%, respectively; *P*<0.01). The time to recurrence at any brain sites (original or distant) was significantly shorter in patients who did not receive postoperative WBRT than in patients who underwent surgery plus WBRT. Another important finding of this study was that the rate of death from neurological causes was significantly higher in the surgery alone group than in the surgery plus WBRT group (44% and 14%, respectively; *P*=0.003). Overall survival time was not significantly different between the two groups (48 weeks in the surgery + WBRT group and 43 weeks in the surgery alone group).

In retrospective cohort studies by Armstrong *et al *[36] and Hagen *et al *[37] comparing patients who underwent surgical resection with those who underwent surgical resection followed by WBRT, similar to the study of Patchell *et al *[35], the overall median survival time did not differ between the two groups. However, in another retrospective study by Skibber *et al *[38], there was a better median survival time in patients who underwent surgery followed by WBRT than in those undergoing surgery alone (18 and six months, respectively; *P*=0.002). The power of this study was low, because only 12 patients were included in the surgery group and 22 patients in the surgery + WBRT group. In another retrospective study from MD Anderson, McPherson *et al *[39] noted that in the study by Patchell and colleagues [35], most patients undergoing WBRT after surgery had relatively radiosensitive primary tumours such as lung and breast cancers, whereas primary cancers that are more resistant to radiation, such as melanoma and renal cell carcinoma, were overlooked [35, 40].

To address this issue, McPherson *et al *[39] have analysed 358 patients with single brain metastases from a wide range of primary tumours, in terms of local tumour control and survival. All patients were treated with microsurgical resection with or without adjuvant WBRT (40% of the patients in the surgery + WBRT group and 60% in the surgery alone group). In the Kaplan–Meier analysis, the median survival time was 14.7 months for the surgery + WBRT group and 11.7 for the surgery alone group (*P*=0.02). Adjuvant WBRT was found to be consistently protective for radiosensitive and radioresistant tumour groups, when each was analysed separately. Nonetheless, in multivariate analysis, WBRT was not a significant independent predictor of survival. Significant predictors for shorter survival were found to be age ≥55 years, evidence of systemic disease, radioresistant tumour histology (primarily melanoma), infratentorial tumour location, interval of <6 months from primary cancer diagnosis to brain tumour diagnosis and preoperative tumour size >3-cm in maximum diameter. Local tumour recurrence rate was 20% for the surgery + WBRT group and 27% for the surgery alone group. The median local progression-free survival time was 14.8 months in the surgery + WBRT group and 9.2 months in the surgery-only group (*P*=0.04). Being at least 55 years old and not undergoing WBRT were associated with local recurrence in the multivariate analysis. Distant recurrence was observed in 30% of patients who underwent surgery + WBRT and in 53% of patients who underwent surgery alone. In the multivariate analysis, withholding WBRT and the presence of a radioresistant tumour type (mainly melanoma) instead of a radiosensitive tumour type (mainly lung, breast and gastrointestinal cancers) was found to be significantly associated with distant tumour recurrence.

In light of these data from the literature, the role of WBRT after surgical resection of a single metastasis is well established in controlling local or distant recurrence, despite the fact that no survival benefit has been shown for it. However, if deterioration of the patient’s neurocognitive functions is a concern, WBRT may be withheld after resection of the lesion (mainly for radioresistant tumours), and WBRT can be used as a salvage therapy.

## Patient Selection/Prognostic Factors

The overall median survival time of patients with brain metastases is poor, and almost half of these patients die from advanced systemic cancer [41, 42]. Therefore, the basic goal of the treatment of brain metastases is to achieve local tumour control and to prevent deaths from neurological causes. More aggressive treatments provide a survival benefit in only a minority of the patients. Prediction of a patient’s prognosis may allow the clinician to tailor the treatment plan; for example, more intensive treatments can be given when they are likely to have a positive effect on survival, or symptom control, whereas disease stabilisation and minimisation of toxicity might be recommended for patients who have more advanced disease and comorbidities that limit an aggressive therapy [41]. Also, prediction of the outcome helps to avoid overtreatment of these patients. Although the role of surgery has been very well described in the literature, response to the treatment may differ among patients, and surgical candidates should be carefully selected.

The characteristics of both the patients and their tumours have been investigated for their prognostic significance in patients with brain metastases. These factors include age, patient’s functional status (mainly evaluated by KPS score), status of primary cancer, activity of systemic disease, neurocognitive function, number of brain metastases, histology of the primary tumour, and interval between the initial cancer diagnosis and detection of brain metastases [43, 44]. Being in good neurological condition or having a high functional status (KPS score >70), a younger age, a controlled primary tumour, the absence of extracranial metastases, and the presence of a solitary brain metastasis are considered to be favourable prognostic factors for survival [41, 44]. Also, a longer interval between the diagnosis of a primary cancer and the occurrence of brain metastasis is associated with longer survival time [44–46]. Conversely, a poorer survival was found when the diagnosis of brain metastasis was within one year of the primary cancer diagnosis [47]. Among these prognostic factors, the KPS score has been shown to be the major predictor for survival; a postoperative KPS score of 70 or more portends the best clinical outcome [48]. Hall *et al *[49] evaluated the long-term survivors from brain metastases and found that after two years, the patients with brain metastases from ovarian cancer had the largest percentage surviving (23.9%), whereas those from small-cell lung carcinoma had the smallest survival percentage (1.7% at two years). In their multivariate analysis, they found that being younger, having a single metastasis, and undergoing surgical resection, WBRT, or chemotherapy were favourable factors for long-term survival.

Based on variables considered to be the best predictors of survival, prognostic indices have been developed. The most widely used prognostic index during the last decade is the recursive partitioning analysis (RPA). RPA is a three-tiered scoring system that was developed in a study of 1200 patients who underwent WBRT during three prospective trials conducted by the RTOG [43]. In these patients, KPS score, age, activity of primary cancer, and status of extracranial disease were assessed to establish the RPA index. RPA class I included those with a KPS score ≥ 70, who were < 65 years old, with a controlled primary tumour, and no extracranial metastases. RPA class III included those with a KPS score < 70, and RPA class II included all other patients. The median survival times for patients in classes I, II, and III were 7.1, 4.2, and 2.3 months, respectively.

This index was validated in two retrospective studies for patients undergoing surgical resection of brain metastases [50, 51]. Agboola and colleagues [50] assessed 125 patients with one or more brain metastases, who underwent resection followed by WBRT, and showed median survival times of 14.8, 9.9 and 6 months for RPA classes I, II, and III, respectively. Conversely, Regine *et al *[51], in a smaller retrospective cohort of 95 patients with a single brain metastasis, did not find any survival difference between RPA classes I and II (10.9 and 9.8 months, respectively). Of note, these authors did not include any RPA class III patients in their study. Tendulkar *et al *[52] retrospectively evaluated 311 patients who had a single brain metastasis. In the univariate analysis, significant variables for longer survival in patients were an age of <65 years, absence of extracranial metastases, controlled primary cancer, RPA class I, and undergoing SRS. Their multivariate analysis showed that an age of <65 years, absence of extracranial metastasis, controlled primary cancer, a histology of non-small cell lung cancer, and undergoing SRS were significant factors for improved survival. The median survival times of patients were 21.4, 9, and 8.9 months for RPA classes I, II, and III, respectively.

Although RPA was developed for patients who received radiation therapy for brain metastases, its prognostic value was also validated for surgical patients in retrospective studies. Yet there is no prognostic index for evaluating surgical patients specifically, and a prognostic grading system may be warranted to better assess patients as candidates for surgical resection of brain metastases.

## Surgical Technique

The main purpose for performing surgical resection of brain metastases is to achieve a gross-total resection of the lesion while protecting the normal functional brain tissue, in order to avoid new neurologic deficits. The following points are important during surgical resection of brain metastases.

### a. Extent of tumour resection

A metastatic lesion is often a well-circumscribed mass with a capsule, which in general displaces, but does not invade, the normal brain. This allows gross-total resection of the tumour without disruption of the tumour capsule. Tendulkar *et al *[52] studied extent of tumour resection and survival in patients with brain metastases and found that the median survival time was better in the gross-total resection group than in the subtotal resection group (10.6 and 8.7 months, respectively; *P*=0.07), although the difference was not statistically significant. This paralleled the earlier findings of Agboola *et al *[50], who demonstrated an increase in survival time of two months in patients who underwent complete resection relative to those who underwent incomplete resection. Two retrospective studies from MD Anderson that analysed the risk of developing leptomeningeal disease for supratentorial and infratentorial single metastases after surgical resection, showed a significantly increased risk of leptomeningeal disease in patients who underwent piecemeal tumour resection compared with en bloc resection [53, 54]. Another study from the same group reported a higher risk of local tumour recurrence with piecemeal resection than with en bloc resection [55]. These data suggest that en bloc resection, if technically possible, prevents dissemination of tumour cells that cause local recurrence of tumours and the appearance of leptomeningeal disease.

### b. Intraoperative image guidance

With the advances in intraoperative imaging technologies, the use of image-guided surgery, functional neuronavigation, and intraoperative ultrasonography has become standard at many institutions [56, 57]. Intraoperative image guidance provides accuracy for targeting the specific lesion and obtaining the appropriate information about the margins of the tumour. Functional navigation helps the neurosurgeon to know the spatial relationship between the lesion and eloquent brain areas and to avoid harming functional brain and producing postoperative deficits. These technologies have become very important for planning the surgical approach and for safety of the surgery. Schackert *et al *[58] has assessed the role of image-guided surgery retrospectively in a cohort of 104 patients with a single brain metastasis. They showed an improvement in KPS score of 10 after surgical resection, despite no survival benefit being evident. A study by Tan and Black [59] analyzed the outcomes of patients after surgical resection that employed image guidance. They found no perioperative mortality, and among 51 symptomatic patients, 70% had complete resolution of their symptoms, 14% had symptom improvement, 12% had no change and 4% showed deterioration. Local tumour control was 84%, and the median survival time was 16.23 months. They concluded that gross-total resection of brain metastases involving eloquent brain areas can be safely performed with a low morbidity rate using intraoperative image guidance and that surgical resection leads to an immediate improvement in neurologic symptoms.

Intraoperative ultrasound is a very useful and inexpensive tool that provides valuable intraoperative real-time information for identifying tumour margins, for showing the relationship between the tumour and critical brain structures, for understanding the components of the lesion (i.e., cystic or solid), and for assessing the extent of tumour resection, which facilitates further resection of the residual tumour [60, 61]. Another intraoperative imaging modality, intraoperative magnetic resonance (iMRI), widely used for infiltrative glial tumours [62, 63], can also be used during resection of metastatic brain tumours if they are large and in deep locations [64]. Of note, iMRI is not often used for resection of brain metastases in our practice at M.D Anderson.

### c. Intraoperative monitoring

Intraoperative monitoring, which includes obtaining somatosensory evoked potentials, motor evoked potentials, and direct cortical stimulation, is another important surgical adjunct for tumour resection. These techniques provide information about the functional cortical surface (such as sensory and motor cortices), deep functional areas (such as the corticospinal tract) [65], and are very useful during surgery in patients harbouring metastatic brain tumours near or within eloquent brain areas. Awake craniotomies are primarily performed in patients with infiltrative tumours such as gliomas; nevertheless, they may be an option for patients whose metastatic brain lesion is in or very close to the speech or motor centres, allowing identification of these critical locations intraoperatively.

## Complications from Surgical Resection

Different series have reported overall postoperative complication rates ranging from ≤5% to 40% in patients who underwent surgical resection for brain metastases [7, 11, 12, 22, 26, 66, 67], and it is important to distinguish between transient and permanent complications. The average major neurological deficit rate reported within 30 days after surgery in a series of 194 patients undergoing resection of brain metastases at MD Anderson was 6% [68]. The study by Muacevic *et al *[32], comparing effectiveness of surgical resection + WBRT with SRS alone for patients with brain metastases, showed a higher incidence of mild complications in the surgery group than in the SRS group but similar rates of more severe complications in both groups. However, Tan and Black [59] demonstrated that resection of brain metastases can be performed safely with low morbidity, even for tumours located in eloquent brain areas.

## Conclusion

Surgical resection has remained the mainstay of treatment for patients with metastatic brain tumours over the last two decades. With technological innovations, new surgical techniques and surgical adjuncts will help neurosurgeons perform more aggressive and safer surgical resections. Finally, it is critical to perform surgery in select patients in whom a benefit from surgical resection will be expected.

## Figures and Tables

**Table 1:** Randomised clinical trials comparing WBRT with and without surgery to treat brain metastases.

Study	Treatment	Number of patients	Median survival time (months)	*P*	Local recurrence
Patchell *et al* [11]	WBRT	23	3.5	<0.01	52%
	WBRT+ surgery	25	9.2		20%
Vecht *et al* [12]	WBRT	31	6	0.04	N/A
	WBRT+ surgery	32	10		N/A

WBRT, whole-brain radiation therapy

**Table 2:** Karnofsky Performance Scale score.

100	Normal; no complaints; no evidence of disease
90	Able to carry on normal activity; minor signs or symptoms of disease
80	Normal activity with effort; some signs or symptoms of disease
70	Cares for self; unable to carry on normal activity or to do active work
60	Requires occasional assistance but is able to care for most personal needs
50	Requires considerable assistance and frequent medical care
40	Disabled; requires special care and assistance
30	Severely disabled; hospitalisation is indicated, although death not imminent
20	Very sick; hospitalisation necessary; active support treatment is necessary
10	Moribund; fatal processes
0	Dead

Source: Ref. [27].
